# Traveling wave solutions of the time-delayed generalized Burgers-type equations

**DOI:** 10.1186/s40064-016-3765-1

**Published:** 2016-12-12

**Authors:** Bo Tang, Yingzhe Fan, Xuemin Wang, Jixiu Wang, Shijun Chen

**Affiliations:** 1 School of Mathematics and Computer Science, Hubei University of Arts and Science, Xiangyang, 441053 China; 2 School of Mathematics and Statistics, Xi’an Jiaotong University, Xi’an, 710049 China; 3 School of Mathematics and Statistics, Wuhan University, Wuhan, 430072 China; 4 Department of Mechanical Engineering, University of Texas at Dallas, Richardson, TX 75080 USA

**Keywords:** Nonlinear time-delayed evolution equations, Extended $\left( \frac{G^{\prime}}{G}\right)$-expansion method, Traveling wave solution

## Abstract

**Background:**

Recently, nonlinear time-delayed evolution equations have received considerable interest due to their numerous applications in the areas of physics, biology, chemistry and so on.

**Methods:**

In this paper, we obtain traveling wave solutions by using the extended $\left( \frac{G^{\prime}}{G}\right)$-expansion method.

**Results:**

Based on the method, we get many solutions of the time-delayed generalized Burgers-type equations.

**Conclusions:**

The results reveal that the extended $\left( \frac{G^{\prime}}{G}\right)$-expansion method is direct, effective and can be used for many other nonlinear time-delayed evolution equations.

## Background

In recent years, theory and numerical analysis of nonlinear time-delayed evolution equations have received considerable interest due to their numerous applications in the areas of physics, biology, chemistry and so on. For better studying the nonlinear physical phenomena of nonlinear time-delayed evolution equations, the solution is much involved. In the past, several analytical and numerical methods have been used to find solutions of nonlinear partial differential equations, such as homotopy perturbation method (Kumar and Singh 2009; Kumar et al. 2012; He 1999), Laplace transform (Kumar 2014), variational iteration method (He 1997; He and Wu 2007; Tang et al. 2014), residual power series method (RPSM for short) (Kumar et al. 2016b; Yao et al. 2015), auxiliary equation method (Sirendaoreji 2003; Tang et al. 2016; Yomba 2004), homotopy analysis method (Yin et al. 2015; Kumar et al. 2016a), $\left( \frac{G^{\prime}}{G}\right)$-expansion method (Wang et al. 2008; Zhang et al. 2010; Tang et al. 2011; Islam et al. 2014; Khan and Akbar 2014) and so on.

In this paper, we apply the extended $\left( \frac{G^{\prime}}{G}\right)$-expansion method to obtain traveling wave solutions of the following time-delayed generalized Burgers-type equations (Kar et al. 2003):

- The time-delayed generalized Burgers equation:
  $$\tau v_{tt}+v_{t}+pv^{s}v_{x}-v_{xx}=0.$$
  where *p*, *s* are constants and $\tau$is a time-delayed constant.
- The time-delayed generalized Burgers-Fisher equation:
  $$\tau v_{tt}+(1-\tau f_{v})v_{t}=v_{xx}-pv^{s}v_{x}+f(v),\quad f(v)=qv(1-v^{s}).$$

This paper is organized as follows: in “Methods” section, the main steps of extended $\left( \frac{G^{\prime}}{G}\right)$-expansion method for obtaining traveling wave solutions of nonlinear time-delayed evolution equation are given. In “Results” section, we construct traveling solutions of the time-delayed generalized Burgers-type equation. Some conclusions are given in “Conclusions” section.

## Methods

Considering the following nonlinear evolution equation:

1 $$P(v, v_{t}, v_{x_{1}}, v_{x_{2}}, v_{x_{3}}, \ldots )=0,$$

where *P* is a polynomial in $v=v(x_{1}, x_{2}, x_{3}, \ldots , t)$ and its various partial derivatives.

### **Step 1**

 By means of the traveling wave transformation

2 $$v=V(\eta ),\qquad \eta =k_{1}x_{1}+k_{2}x_{2}+k_{3}x_{3}+\cdots +ht+\eta _{0},$$

where the coefficients $k_{i}$, *h* are constants. Equation (1) can be transformated as follows:

3 $$P(V(\eta ), V^{\prime}(\eta ), V^{^{\prime\prime}}(\eta ),\ldots )=0.$$

### **Step 2**

 We suppose that the Eq. (3) has the following solution:

4 $$V(\eta )=\sum _{l=-n}^{n}a_{l}\left( \frac{G^{\prime}}{G}\right) ^{l},$$

where $a_{l}$ are constants to be determined later, and $G(\eta )$ satisfies the following equation:

5 $$G^{\prime\prime}(\eta )+\alpha G^{\prime}(\eta )+\beta G(\eta )=0,$$

where $\alpha$ and $\beta$ are arbitrary constants. Based on Eq. (5), we have

$$\frac{G^{\prime}(\eta )}{G(\eta )}= \left\{ \begin{array}{ll} -\frac{\alpha }{2}+\frac{\sqrt{\alpha ^{2}-4\beta }}{2} \left( \frac{C_{1}\sinh \left(\frac{\sqrt{\alpha ^{2}-4\beta }\eta }{2}\right)+C_{2}\cosh \left(\frac{\sqrt{\alpha ^{2}-4\beta }\eta }{2}\right)}{C_{1}\cosh \left(\frac{\sqrt{\alpha ^{2}-4\beta }\eta }{2}\right)+C_{2}\sinh \left(\frac{\sqrt{\alpha ^{2}-4\beta }\eta }{2}\right)}\right) ,&\quad\alpha ^{2}-4\beta >0, \\ -\frac{\alpha }{2}+\frac{\sqrt{4\beta -\alpha ^{2}}}{2} \left( \frac{-C_{1}\sin \left(\frac{\sqrt{4\beta -\alpha ^{2}}\eta }{2}\right)+C_{2}\cos \left(\frac{\sqrt{4\beta -\alpha ^{2}}\eta }{2}\right)}{C_{1}\cos \left(\frac{\sqrt{4\beta -\alpha ^{2}}\eta }{2}\right)+C_{2}\sin \left(\frac{\sqrt{4\beta -\alpha ^{2}}\eta }{2}\right)}\right) ,&\quad\alpha ^{2}-4\beta <0.\\ \frac{C_{2}}{C_{1}+C_{2}\eta }-\frac{\alpha }{2}, &\quad \alpha ^{2}-4\beta =0. \end{array} \right.$$

### **Step 3**

Determine the degree *n* in Eq. (3) by use of homogenous balanced principle (Abdel Rady et al. 2010; Fan and Zhang 1998a, b; Senthilvelan 2001; Zhao and Tang 2002; Fan 2000; Eslami et al. 2014), namely balancing the highest order derivatives and nonlinear terms in Eq. (3).

### **Step 4**

 Substituting Eqs. (4) and (5) into Eq. (3) and clearing the denominator and collecting all terms with the same order of $\left( \frac{G^{\prime}}{G}\right)$ together, then setting each coefficient of $\left( \frac{G^{\prime}}{G}\right) ^{l}$ to zero, we get a system of under-determined algebraic equations for $k_{i}, h$ and $a_{l}$.

### **Step 5**

Solving the algebraic equations in Step 4 by Maple (www.maplesoft.com), we can finally get traveling wave solutions of Eq. (1).

## Results

In this section, we apply the extended $\left( \frac{G^{\prime}}{G}\right)$-expansion method to obtain traveling wave solutions of the time-delayed generalized Burgers-type equations.

### Solutions to the time-delayed generalized Burgers equation

We consider the following time-delayed generalized Burgers equation:

6 $$\tau v_{tt}+v_{t}+pv^{s}v_{x}-v_{xx}=0.$$

By using transformations $v(x, t)=V(\eta )$ and $\eta =k(x-\omega t)$, Eq. (6) can be reduced as follows:

7 $$(\tau \omega ^{2}-1)k^{2}V^{\prime\prime}-k\omega V^{\prime}+pkV^{s}V^{\prime}=0.$$

Balancing $V^{\prime\prime}$ with $V^{s}V^{\prime}$ gives $n=\frac{1}{s}$ which is not an integer as $s\ne 1$. So we use a transformation $V=W^{\frac{1}{s}}$ to change Eq. (7) into the form:

8 $$(\tau \omega ^{2}-1)k^{2}\left[W^{\prime\prime}W+\left(\frac{1}{s}-1\right)W^{\prime 2}\right]-k\omega W^{\prime}W+pkW^{\prime}W^{2}=0.$$

We suppose that the solutions of (8) have the form (4) and (5), so

$$\begin{aligned} W^{\prime}(\eta )= &\, \sum _{l=-n}^{n} a_{l}\left( \frac{G^{\prime}}{G}\right) ^{l-1}\frac{G^{\prime\prime}G-G^{\prime 2}}{G^{2}}\\= &\, -\sum _{l=-n}^{n} a_{l}\left( \frac{G^{\prime}}{G}\right) ^{l-1}\left[\beta +\alpha \left( \frac{G^{\prime}}{G}\right) +\left( \frac{G^{\prime}}{G}\right) ^{2}\right],\\ W^{\prime\prime}(\eta )= &\, \sum _{l=-n}^{n} a_{l}\left( \frac{G^{\prime}}{G}\right) ^{l-2}\left[\beta +\alpha \left( \frac{G^{\prime}}{G}\right) +\left( \frac{G^{\prime}}{G}\right) ^{2}\right]^{2}\\&+\sum _{l=-n}^{n} a_{l}\left( \frac{G^{\prime}}{G}\right) ^{l-1}\left[\alpha +2\left( \frac{G^{\prime}}{G}\right) \right]\left[\beta +\alpha \left( \frac{G^{\prime}}{G}\right) +\left( \frac{G^{\prime}}{G}\right) ^{2}\right]. \end{aligned}$$

From above two equations, we can get the degrees of $W^{\prime\prime}W$ and $W^{\prime}W^{2}$ are $2n+2$ and $3n+1$ respectively. Balancing $W^{\prime\prime}W$ and $W^{\prime}W^{2}$ in Eq. (8) yields $2n+2=3n+1$, namely $n=1$. Therefore Eq. (8) have the following solutions:

9 $$W(\eta )=\sum _{l=-1}^{1} a_{l}\left( \frac{G^{\prime}}{G}\right) ^{l}.$$

Substituting Eqs. (9) and (5) into Eq. (8), we get a set of under-determined algebraic equations for $a_{l}(l=0,\pm 1)$, $k, \omega , \alpha$ and $\beta$.

$$\begin{aligned} \left( \frac{G^{\prime}}{G}\right) ^{4}:&\,2(\tau \omega ^{2}-1)ka_{1}^{2}+\frac{k(\tau \omega ^{2}-1)(1-s)a_{1}^{2}}{s}-pa_{1}^{3}=0,\\ \left( \frac{G^{\prime}}{G}\right) ^{3}:&\,3(\tau \omega ^{2}-1)k\alpha a_{1}^{2}+2(\tau \omega ^{2}-1)ka_{1}a_{0}\\&+\,\frac{2k\alpha (\tau \omega ^{2}-1)(1-s)a_{1}^{2}}{s}+\omega a_{1}^{2}-2pa_{0}a_{1}^{2}-p\alpha a_{1}^{3}=0,\\ \left( \frac{G^{\prime}}{G}\right) ^{2}:&\,\frac{(\tau \omega ^{2}-1)k(1-s)(-2a_{1}a_{-1}+2a_{1}^{2}\beta + a_{1}^{2}\alpha ^{2})}{s}+pa_{0}^{2}a_{1}(1+2\alpha )\\&+\,(\tau \omega ^{2}-1)k(\alpha ^{2}+2\beta )a_{1}^{2}+3(\tau \omega ^{2}-1)k\alpha a_{1}a_{0}-pa_{1}^{3}\beta \\&+\,2(\tau \omega ^{2}-1)ka_{1}a_{-1}+\omega a_{0}a_{1}+\omega a_{1}^{2}\alpha -pa_{1}^{2}a_{-1}=0,\\ \left( \frac{G^{\prime}}{G}\right) ^{1}:&\,\frac{(\tau \omega ^{2}-1)k(1-s)(-4a_{-1}+2a_{1}\beta )a_{1}\alpha }{s}-pa_{1}\alpha (a_{1}a_{-1}+a_{0}^{2})\\&+\,(\tau \omega ^{2}-1)ka_{1}(a_{1}\alpha \beta +4a_{-1}\alpha +a_{0}\alpha ^{2}+2a_{0}\beta )\\&-\,2pa_{0}a_{1}^{2}\beta +\omega a_{1}(a_{0}\alpha +a_{1}\beta )=0,\\ \left( \frac{G^{\prime}}{G}\right) ^{0}:&\,(\tau \omega ^{2}-1)k(4a_{-1}a_{1}\beta +2a_{-1}a_{1}\alpha ^{2}+a_{0}a_{1}\beta \alpha +a_{-1}a_{0}\alpha )\\&+\,(a_{-1}-a_{1}\beta )(a_{-1}a_{1}+a_{0}^{2})p-\omega a_{0}a_{-1}-\omega a_{0}a_{1}\beta \\&+\,\frac{(\tau \omega ^{2}-1)k(1-s)}{s}(-4a_{-1}a_{1}\beta -2a_{-1}a_{1}\alpha ^{2}+a_{-1}^{2}+a_{1}^{2}\beta ^{2})=0,\\ \left( \frac{G^{\prime}}{G}\right) ^{-1}:&\,(\tau \omega ^{2}-1)k(4a_{-1}a_{1}\alpha \beta +2a_{-1}a_{0}\beta +a_{-1}a_{0}\alpha ^{2}+a_{-1}^{2}\alpha )\\&-\,\omega a_{-1}(a_{-1}+a_{0}\alpha )+pa_{-1}^{2}a_{1}\alpha +pa_{-1}a_{0}(2a_{-1}+a_{0}\alpha )\\&+\,\frac{(\tau \omega ^{2}-1)k\alpha (1-s)}{s}(-4a_{-1}a_{1} \beta +2a_{-1}^{2})=0,\\ \left( \frac{G^{\prime}}{G}\right) ^{-2}:&\,\frac{(\tau \omega ^{2}-1)ka_{-1}(1-s)}{s}(2a_{-1}\beta -2a_{1}\beta ^{2}+a_{-1}\alpha ^{2})\\&-\,\omega a_{-1}(a_{-1}\alpha +a_{0}\beta )+pa_{-1}^{2}(2a_{0}\alpha +a_{1}\beta )+pa_{-1}^{3}+pa_{0}^{2}a_{-1}\beta \\&+\,(\tau \omega ^{2}-1)k(2a_{-1}a_{1}\beta ^{2}+3a_{-1}a_{0}\alpha \beta +2a_{-1}^{2}\mu +a_{-1}^{2}\alpha ^{2})=0,\\ \left( \frac{G^{\prime}}{G}\right) ^{-3}:&\,\frac{2(\tau \omega ^{2}-1)ka_{-1}^{2}\alpha \beta (1-s)}{s}+(\tau \omega ^{2}-1)ka_{-1}\beta (2a_{0}\beta +3a_{-1}\alpha )\\&-\,\omega a_{-1}^{2}\beta +pa_{-1}^{2}(a_{-1}\alpha +2a_{0}\beta )=0,\\ \left( \frac{G^{\prime}}{G}\right) ^{-4}:&\,\frac{(\tau \omega ^{2}-1)ka_{-1}^{2}\beta ^{2}(1-s)}{s}+2(\tau \omega ^{2}-1)ka_{-1}^{2}\beta ^{2}+pa_{-1}^{3}\beta =0. \end{aligned}$$

Solving this algebraic equations by Maple, we can obtain the two results:

#### **Case 1**

10 $$\begin{aligned} &a_{-1}= \pm \frac{(s+1)\beta \omega }{p\sqrt{\alpha ^{2}-4\beta }},\quad a_{0}=\pm \frac{\alpha (s+1)\omega }{2p\sqrt{\alpha ^{2}-4\beta }}+\frac{(s+1)\omega }{2p}, \\ & a_{1}= 0,\quad k=\mp \frac{s\omega }{(\tau \omega ^{2}-1)\sqrt{\alpha ^{2}-4\beta }}, \end{aligned}$$

where $\alpha$, $\beta$ and $\omega$ are arbitrary constants.

#### **Case 2**

11 $$\begin{aligned} &a_{-1}= 0,\quad a_{0}=\pm \frac{\alpha (s+1)\omega }{2p\sqrt{\alpha ^{2}-4\beta }}+\frac{(s+1)\omega }{2p}, \\ & a_{1}= \pm \frac{(s+1)\omega }{p\sqrt{\alpha ^{2}-4\beta }},\quad k=\pm \frac{s\omega }{(\tau \omega ^{2}-1)\sqrt{\alpha ^{2}-4\beta }}, \end{aligned}$$

where $\alpha$, $\beta$ and $\omega$ are arbitrary constants.

Using Eqs. (9) and (10), we obtain the following solution of Eq. (6):

12 $$v_{1}(\eta )=\left[\pm \frac{\alpha (s+1)\omega }{2p\sqrt{\alpha ^{2}-4\beta }}+\frac{(s+1)\omega }{2p}\pm \frac{(s+1)\beta \omega }{p\sqrt{\alpha ^{2}-4\beta }}\left( \frac{G^{\prime}}{G}\right) ^{-1}\right]^{\frac{1}{s}},$$

where $$\eta =\mp \frac{s\omega }{(\tau \omega ^{2}-1)\sqrt{\alpha ^{2}-4\beta }}\,(x-\omega t)$$.

Based on Eqs. (9) and (11), we get the solution of Eq. (6) as follows:

13 $$v_{2}(\eta )=\left[\pm \frac{\alpha (s+1)\omega }{2p\sqrt{\alpha ^{2}-4\beta }}+\frac{(s+1)\omega }{2p}\pm \frac{(s+1)\omega }{p\sqrt{\alpha ^{2}-4\beta }}\left( \frac{G^{\prime}}{G}\right) \right]^{\frac{1}{s}},$$

where $$\eta =\pm \frac{s\omega }{(\tau \omega ^{2}-1)\sqrt{\alpha ^{2}-4\beta }}\,(x-\omega t)$$.

Substituting the general solutions of Eq. (5) into Eq. (12), we have two kinds of travelling wave solutions as follows:

When $$\alpha ^{2}-4\beta >0$$,

14 $$v(x, t)=\left[\frac{(s+1)\omega }{p\sqrt{\alpha ^{2}-4\beta }}\left(\frac{\sqrt{\alpha ^{2}-4\beta }\pm \alpha }{2} \pm \frac{2\beta }{-\alpha +\sqrt{\alpha ^{2}-4\beta } \left(\frac{C_{1}\sinh \frac{\sqrt{\alpha ^{2}-4\beta }\eta }{2}+C_{2}\cosh \frac{\sqrt{\alpha ^{2}-4\beta }\eta }{2}}{C_{1}\cosh \frac{\sqrt{\alpha ^{2}-4\beta }\eta }{2}+C_{2}\sinh \frac{\sqrt{\alpha ^{2}-4\beta }\eta }{2}}\right)}\right)\right]^{\frac{1}{s}},$$

where $$\eta =\mp \frac{s\omega }{(\tau \omega ^{2}-1)\sqrt{\alpha ^{2}-4\beta }}\,(x-\omega t)$$.

When $$\alpha ^{2}-4\beta <0$$,

15 $$v(x,t)=\left[\frac{(s+1)\omega }{p\sqrt{\alpha ^{2}-4\beta }}\left(\frac{\sqrt{\alpha ^{2}-4\beta }\pm \alpha }{2}\pm\frac{2\beta }{-\alpha +\sqrt{4\beta -\alpha ^{2}}\left(\frac{-C_{1}\sin \frac{\sqrt{4\beta -\alpha ^{2}}\eta }{2}+C_{2}\cos \frac{\sqrt{4\beta -\alpha ^{2}}\eta }{2}}{C_{1}\cos \frac{\sqrt{4\beta -\alpha ^{2}}\eta }{2}+C_{2}\sin \frac{\sqrt{4\beta -\alpha ^{2}}\eta }{2}}\right)}\right)\right]^{\frac{1}{s}},$$

where $$\eta =\mp \frac{s\omega }{(\tau \omega ^{2}-1)\sqrt{\alpha ^{2}-4\beta }}\,(x-\omega t)$$.

Substituting the general solutions of Eq. (5) into Eq. (13), we have the following two kinds of travelling wave solutions:

When $$\alpha ^{2}-4\beta >0$$,

16 $$u(x, t)=\left[\frac{(s+1)\omega }{2p}\pm \frac{(s+1)\omega }{2p} \left(\frac{C_{1}\sinh \frac{\sqrt{\alpha ^{2}-4\beta }\eta }{2}+C_{2}\cosh \frac{\sqrt{\alpha ^{2}-4\beta }\eta }{2}}{C_{1}\cosh \frac{\sqrt{\alpha ^{2}-4\beta }\eta }{2}+C_{2}\sinh \frac{\sqrt{\alpha ^{2}-4\beta }\eta }{2}}\right)\right]^{\frac{1}{s}}$$

where $$\eta =\pm \frac{s\omega }{(\tau \omega ^{2}-1)\sqrt{\alpha ^{2}-4\beta }}\,(x-\omega t)$$.

When $$\alpha ^{2}-4\beta <0$$,

17 $$u(x, t)=\left[\frac{(s+1)\omega }{2p}\mp \frac{(s+1)\omega }{2p}i \left(\frac{-C_{1}\sin \frac{\sqrt{4\beta -\alpha ^{2}}\eta }{2}+C_{2}\cos \frac{\sqrt{4\beta -\alpha ^{2}}\eta }{2}}{C_{1}\cos \frac{\sqrt{4\beta -\alpha ^{2}}\eta }{2}+C_{2}\sin \frac{\sqrt{4\beta -\alpha ^{2}}\eta }{2}}\right)\right]^{\frac{1}{s}}$$

where $$\eta =\pm \frac{s\omega }{(\tau \omega ^{2}-1)\sqrt{\alpha ^{2}-4\beta }}\,(x-\omega t)$$.

**Fig. 1:**
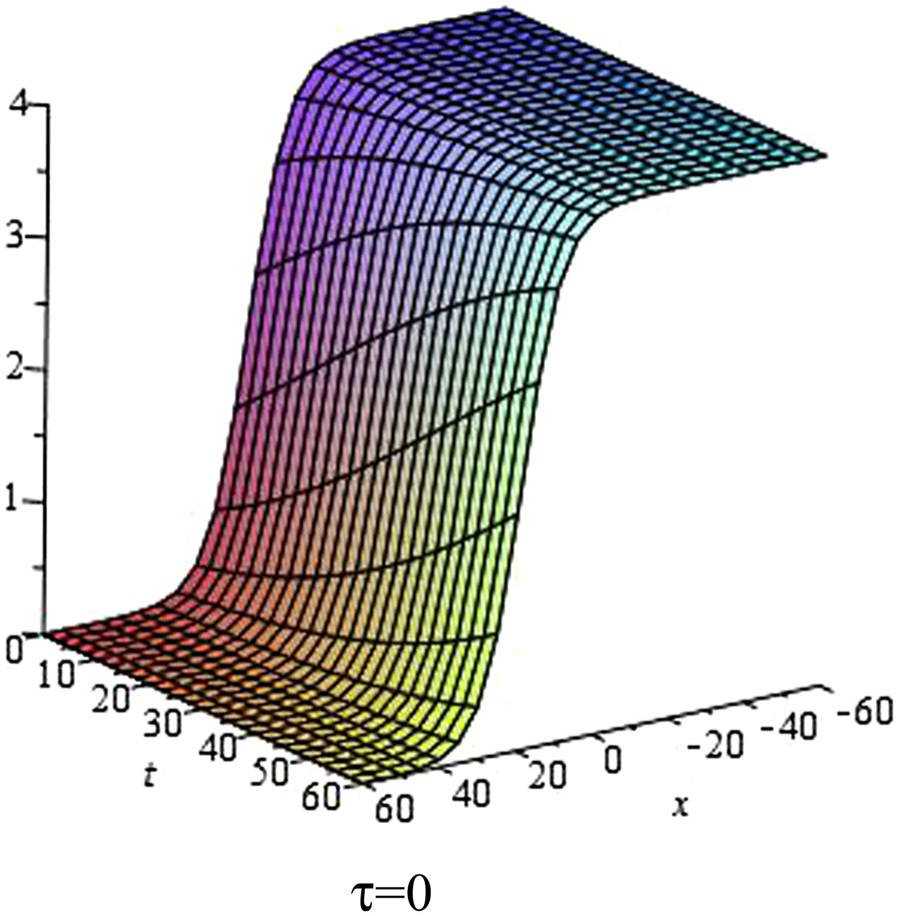
The solution (14) for $\tau = 0$ at $s = 1$, $p = 0.1$, $\omega = 0.2$, $\alpha = 5$, $\beta = 4$, $t = 1$, $C_{2} = 0$

**Fig. 2:**
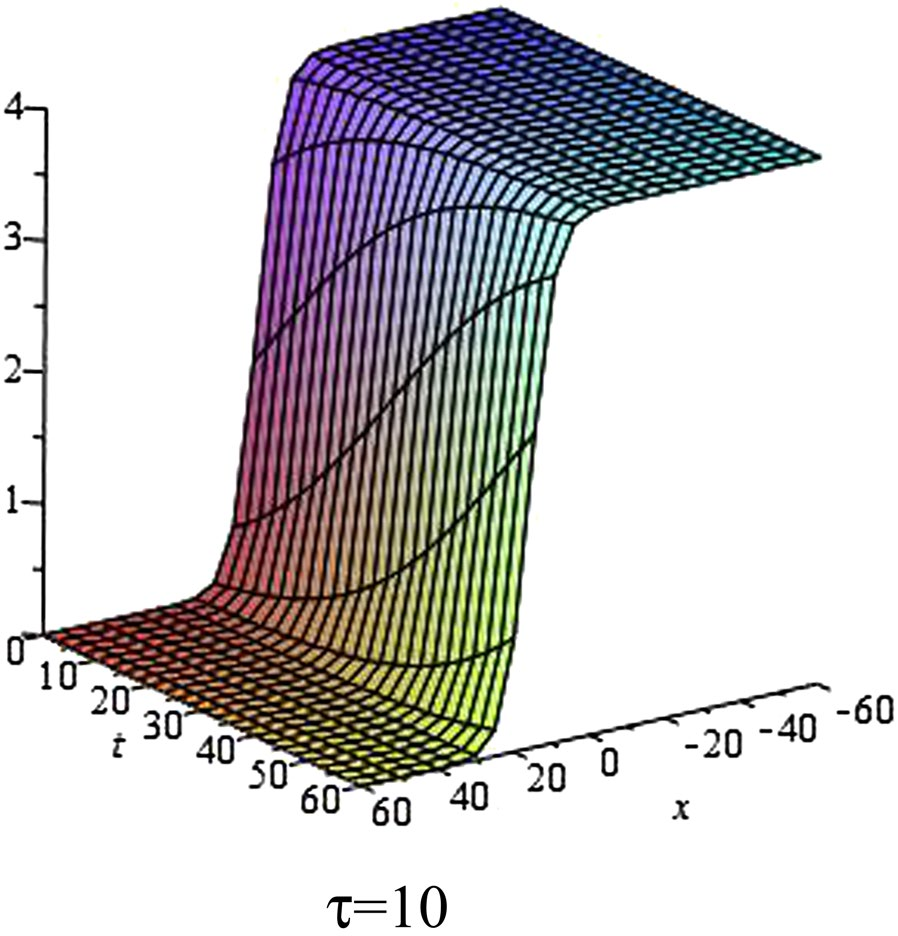
The solution (14) for $\tau = 10$ at $s = 1$, $p = 0.1$, $\omega = 0.2$, $\alpha = 5$, $\beta = 4$, $t = 1$, $C_{2} = 0$

**Fig. 3:**
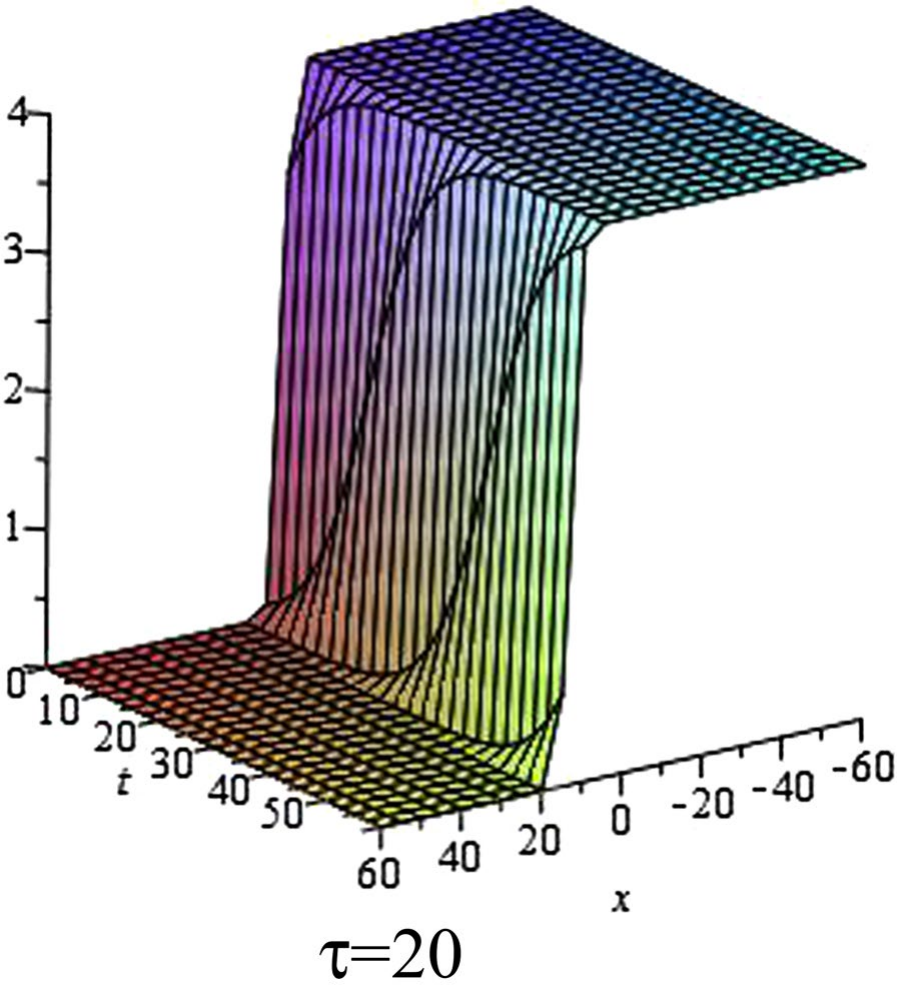
The solution (14) for $\tau = 20$ at $s = 1$, $p = 0.1$, $\omega = 0.2$, $\alpha = 5$, $\beta = 4$, $t = 1$, $C_{2} = 0$

**Fig. 4:**
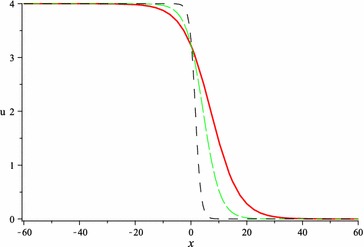
The *red*, *green* and *black lines* represent the solution (14) for $\tau = 0, 10, 20$ respectively at $s = 1$, $p = 0.1$, $\omega = 0.2$, $\alpha = 5$, $\beta = 4$, $t = 1$, $C_{2} = 0$

In Figs. 1, 2, 3 and 4, we show the effect of the time-delayed solution (14). It should be noted that when $\tau \rightarrow 0$, we can recover some traveling wave solutions of the generalized Burgers equation.

### Solutions to the time-delayed generalized Burgers–Fisher equation

In this section, we consider the time-delayed generalized Burgers–Fisher equation:

18 $$\tau v_{tt}+(1-\tau f_{v})v_{t}=v_{xx}-pv^{s}v_{x}+f(v),\quad f(v)=qv(1-v^{s}),\quad q\ne 0.$$

By using the transformation

19 $$v(x, t)=v(\eta ),\quad \eta =k(x-\omega t)$$

Equation (18) is converted into the following ordinary differential equation:

20 $$k^{2}(\tau \omega ^{2}-1)v^{\prime\prime}-k\omega (1-\tau q+(s+1)q v^{s})v^{\prime}+pkv^{s}v^{\prime}-q v(1-v^{s})=0.$$

Balancing $v^{\prime\prime}$ and $v^{s}v^{\prime}$ in Eq. (20) gives $n=\frac{1}{s}$. By using the transformation $v=W^{\frac{1}{s}}$, we convert Eq. (20) into

21 $$\begin{aligned}&k^{2}(\tau \omega ^{2}-1)\left(\frac{1}{s}-1\right)(W^{\prime 2}+W W^{\prime\prime})-k\omega (1-\tau q+(s+1)q W)W W^{\prime} \\ &\quad+\,pkv^{2}W^{\prime}-sq W^{2}(1-W)=0. \end{aligned}$$

By balancing $W^{\prime 2}$ and $W^{2}W^{\prime}$ in Eq. (21), we suppose that Eq. (21) have the following solutions:

22 $$W(\eta )=\sum _{l=-1}^{1} a_{l}\left( \frac{G^{\prime}}{G}\right) ^{l}.$$

Using the same procedure as in the previous example, we get a set of simultaneous algebraic equations for $a_{l}$, $k, \omega , \alpha$ and $\beta$.

$$\begin{aligned} \left( \frac{G^{\prime}}{G}\right) ^{4}:&\,-pka_{1}^{3}+k\omega (s+1)q\tau a_{1}^{3}+(\tau \omega ^{2}-1)k^{2}a_{1}^{2}\left(1+\frac{1}{s}\right)=0,\\ \left( \frac{G^{\prime}}{G}\right) ^{3}:&\,k^{2}(\tau \omega ^{2}-1)\left[2\left(1-\frac{1}{s}\right)\alpha a_{1}+a_{0}\right]a_{1}+k\omega (1-q\tau )a_{1}^{2}\\&+\,(\omega (s+1)-p) k(2a_{0}+\alpha a_{1})a_{1}^{2}+sqa_{1}^{3}=0,\\ \left( \frac{G^{\prime}}{G}\right) ^{2}:&\,\frac{k^{2}(\tau \omega ^{2}-1)a_{1}}{s}((\alpha ^{2}+2\beta )a_{1}+(2s-1)a_{-1}+3a_{0}\alpha s)\\&-\,p ka_{1}(a_{-1}a_{1}+a_{0}+2\alpha a_{0}a_{1}+a_{1}^{2}\beta )+k\omega (1-q\tau )(a_{0}+a_{1}\alpha )a_{1}\\&+\,k\omega (s+1)q\tau a_{1}(a_{0}^{2}+a_{-1}a_{1}+2a_{0}a_{1}\alpha +a_{1}^{2}\beta )+sqa_{1}^{2}(3a_{0}-1)=0,\\ \left( \frac{G^{\prime}}{G}\right) ^{1}:&\,\frac{k^{2}(\tau \omega ^{2}-1)a_{1}}{s}[(8s-1)a_{-1}\alpha +(2-s)a_{1}\beta \alpha +s(\alpha ^{2}+\beta )a_{0}]\\&+\,sqa_{1}(3a_{-1}a_{1}+3a_{0}^{2}-2a_{0})+k\omega (1-q\tau )(a_{0}a_{1}\alpha +a_{1}^{2}\beta )\\&+\,pka_{1}(-a_{-1}a_{1}\alpha -a_{0}^{2}\alpha +2a_{-1}a_{0}-2a_{0}a_{1}\beta )=0,\\ \left( \frac{G^{\prime}}{G}\right) ^{0}:&\,k\omega (s+1)q\tau (a_{-1}a_{1}-a_{0}^{2})(a_{1}\beta -a_{-1})+sq(6a_{-1}a_{0}a_{1}-2a_{-1}a_{1})\\&-\,k\omega (1-q\tau )a_{0}a_{-1}+k\omega (1-q\tau )a_{1}\beta +p k(a_{-1}-a_{1}\beta )(a_{-1}a_{1}+a_{0}^{2})\\&+\,sq(-a_{0}^{2}+a_{0}^{3})+\frac{k^{2}(\tau \omega ^{2}-1)}{s}[4(2s-1)a_{-1}a_{1}\beta +(5s-2)a_{-1}a_{1}\alpha ^{2}\\&+\,(1-s)(a_{-1}^{2}+a_{1}^{2}\beta ^{2})+s\alpha a_{0}(a_{1}\beta +a_{-1})]=0,\\ \left( \frac{G^{\prime}}{G}\right) ^{-1}:&\,-k\omega (1-q\tau )a_{-1}(a_{-1}+a_{0}\alpha )+pka_{-1}(2a_{-1}a_{0}+a_{0}^{2}\alpha +a_{-1}a_{1}\alpha )\\&+\,2k^{2}(\tau \omega ^{2}-1)\alpha a_{-1}\left(\frac{1}{s}-1\right)(a_{-1}-2a_{1}\beta )+sqa_{-1}(3a_{-1}a_{1}-2a_{0}+3a_{0}^{2})\\&+\,k^{2}(\tau \omega ^{2}-1)a_{-1}(4a_{1}\alpha \beta +2a_{0}\beta +a_{0}\alpha ^{2}+a_{-1}\alpha )\\&-\,k\omega (s+1)q\tau a_{-1}(2a_{-1}a_{0}+a_{-1}a_{1}\alpha +a_{0}^{2}\alpha )=0,\\ \left( \frac{G^{\prime}}{G}\right) ^{-2}:&\,pka_{-1}(a_{-1}^{2}+2a_{-1}a_{0}\alpha +a_{-1}a_{1}\beta +a_{0}^{2}\beta )+qsa_{-1}^{2}(3a_{0}-1)\\&-\,k\omega (1-q\tau )a_{-1}(a_{-1}\alpha +a_{0}\beta +a_{-1}^{2}-a_{-1}a_{1}\beta )\\&+\,\frac{k^{2}(\tau \omega ^{2}-1)a_{-1}}{s}(2a_{-1}\beta +2(2s-1)a_{1}\beta ^{2}+a_{-1}\alpha ^{2}+3a_{0}\alpha \beta s)\\&-\,k\omega (s+1)q\tau a_{-1}(2a_{-1}a_{0}\alpha +2a_{-1}a_{1}\beta +a_{0}^{2}\beta )=0,\\ \left( \frac{G^{\prime}}{G}\right) ^{-3}:&\,\frac{k^{2}(\tau \omega ^{2}-1)a_{-1}\beta }{s}((s+2)a_{-1}\alpha +2sa_{0}\beta )+qsa_{-1}^{3}\\&+\,ka_{-1}^{2}(a_{-1}\alpha +2a_{0}\beta )(p-\omega (s+1)q\tau )-k\omega (1-q\tau )a_{-1}^{2}\beta =0,\\ \left( \frac{G^{\prime}}{G}\right) ^{-4}:&\,k^{2}(\tau \omega ^{2}-1)\left(\frac{1}{s}+1\right)a_{-1}^{2}\beta ^{2}+pka_{-1}^{3}\beta -k\omega (s+1)q\tau a_{-1}^{3}\beta =0. \end{aligned}$$

Solving the under-determined algebraic equations, we have the following results:

**Case 1**

23 $$\begin{aligned} &a_{-1}= \pm \frac{\beta }{\sqrt{\alpha ^{2}-4\beta }},\quad a_{0}=\frac{1}{2}\pm \frac{\alpha }{2\sqrt{\alpha ^{2}-4\beta }},\quad a_{1}=0, \\ & k= \mp \frac{s(s+1)(1+q\tau )p}{(\tau p^{2}-(s+1)^{2})\sqrt{\alpha ^{2}-4\beta }},\quad \omega =\frac{p^{2}+(s+1)^{2}q}{p(s+1)(1+q\tau )}. \end{aligned}$$

**Case 2**

24 $$\begin{aligned} &a_{-1}= 0,\quad a_{0}=\frac{1}{2}\pm \frac{\alpha }{2\sqrt{\alpha ^{2}-4\beta }},\quad a_{1}=\pm \frac{1}{\sqrt{\alpha ^{2}-4\beta }}, \\ & k= \pm \frac{s(s+1)(1+q\tau )p}{(\tau p^{2}-(s+1)^{2})\sqrt{\alpha ^{2}-4\beta }},\quad \omega =\frac{p^{2}+(s+1)^{2}q}{p(s+1)(1+q\tau )}. \end{aligned}$$

By using Eqs. (23) and (24), expression (22) can be written as:

25 $$v_{1}(\eta )=\frac{1}{2}\pm \frac{\alpha }{2\sqrt{\alpha ^{2}-4\beta }}\pm \frac{\beta }{\sqrt{\alpha ^{2}-4\beta }}\left( \frac{G^{\prime}}{G}\right) ^{-1},$$

where $$\eta =\mp \frac{s(s+1)(1+q\tau )p}{(\tau p^{2}-(s+1)^{2})\sqrt{\alpha ^{2}-4\beta }}\left(x-\frac{p^{2}+(s+1)^{2}q}{p(s+1)(1+q\tau )}t\right)$$.

26 $$v_{2}(\eta )=\frac{1}{2}\pm \frac{\alpha }{2\sqrt{\alpha ^{2}-4\beta }}\pm \frac{1}{\sqrt{\alpha ^{2}-4\beta }}\left( \frac{G^{\prime}}{G}\right) ,$$

where $$\eta =\pm \frac{s(s+1)(1+q\tau )p}{(\tau p^{2}-(s+1)^{2})\sqrt{\alpha ^{2}-4\beta }}\left(x-\frac{p^{2}t+(s+1)^{2}qt}{p(s+1)(1+q\tau )}\right)$$.

Substituting general solutions of Eq. (5) into Eqs. (25) and (26), we have two types of travelling wave solutions of the generalized time-delayed Burgers-Fisher equation as follows:

When $$\alpha ^{2}-4\beta >0$$,

27 $$u(x, t)=\left[\frac{1}{\sqrt{\alpha ^{2}-4\beta }}\left(\frac{\sqrt{\alpha ^{2}-4\beta }\pm \alpha }{2} \pm\frac{2\beta }{-\alpha +\sqrt{\alpha ^{2}-4\beta } \left(\frac{C_{1}\sinh \frac{\sqrt{\alpha ^{2}-4\beta }\eta }{2}+C_{2}\cosh \frac{\sqrt{\alpha ^{2}-4\beta }\eta }{2}}{C_{1}\cosh \frac{\sqrt{\alpha ^{2}-4\beta }\eta }{2}+C_{2}\sinh \frac{\sqrt{\alpha ^{2}-4\beta }\eta }{2}}\right)}\right)\right]^{\frac{1}{s}},$$

where $$\eta =\mp \frac{s(s+1)(1+q\tau )p}{(\tau p^{2}-(s+1)^{2})\sqrt{\alpha ^{2}-4\beta }}\left(x-\frac{p^{2}t+(s+1)^{2}qt}{p(s+1)(1+q\tau )}\right)$$.

28 $$u(x, t)=\left[\frac{1}{2} \pm \frac{1}{2}\left(\frac{C_{1}\sinh \frac{\sqrt{\alpha ^{2}-4\beta }\eta }{2}+C_{2}\cosh \frac{\sqrt{\alpha ^{2}-4\beta }\eta }{2}}{C_{1}\cosh \frac{\sqrt{\alpha ^{2}-4\beta }\eta }{2}+C_{2}\sinh \frac{\sqrt{\alpha ^{2}-4\beta }\eta }{2}}\right)\right]^{\frac{1}{s}},$$

where $$\eta =\pm \frac{s(s+1)(1+q\tau )p}{(\tau p^{2}-(s+1)^{2})\sqrt{\alpha ^{2}-4\beta }}\left(x-\frac{p^{2}+(s+1)^{2}q}{p(s+1)(1+q\tau )}t\right)$$.

When $$\alpha ^{2}-4\beta <0$$,

29 $$u(x, t)=\left[\frac{1}{\sqrt{\alpha ^{2}-4\beta }}\left(\frac{\sqrt{\alpha ^{2}-4\beta }\pm \alpha }{2} \pm \frac{2\beta }{-\alpha +\sqrt{\alpha ^{2}-4\beta } \left(\frac{-C_{1}\sin \frac{\sqrt{4\beta -\alpha ^{2}}\eta }{2}+C_{2}\cos \frac{\sqrt{4\beta -\alpha ^{2}}\eta }{2}}{C_{1}\cos \frac{\sqrt{4\beta -\alpha ^{2}}\eta }{2}+C_{2}\sin \frac{\sqrt{4\beta -\alpha ^{2}}\eta }{2}}\right)}\right)\right]^{\frac{1}{s}},$$

where $$\eta =\mp \frac{s(s+1)(1+q\tau )p}{(\tau p^{2}-(s+1)^{2})\sqrt{\alpha ^{2}-4\beta }}\left(x-\frac{p^{2}+(s+1)^{2}q}{p(s+1)(1+q\tau )}t\right)$$.

30 $$u(x, t)=\left[\frac{1}{2}\mp \frac{i}{2}\left(\frac{-C_{1}\sin \frac{\sqrt{4\beta -\alpha ^{2}}\eta }{2}+C_{2}\cos \frac{\sqrt{4\beta -\alpha ^{2}}\eta }{2}}{C_{1}\cos \frac{\sqrt{4\beta -\alpha ^{2}}\eta }{2}+C_{2}\sin \frac{\sqrt{4\beta -\alpha ^{2}}\eta }{2}}\right)\right]^{\frac{1}{s}},$$

where $$\eta =\pm \frac{s(s+1)(1+q\tau )p}{(\tau p^{2}-(s+1)^{2})\sqrt{\alpha ^{2}-4\beta }}\left(x-\frac{p^{2}+(s+1)^{2}q}{p(s+1)(1+q\tau )}t\right)$$.

**Fig. 5:**
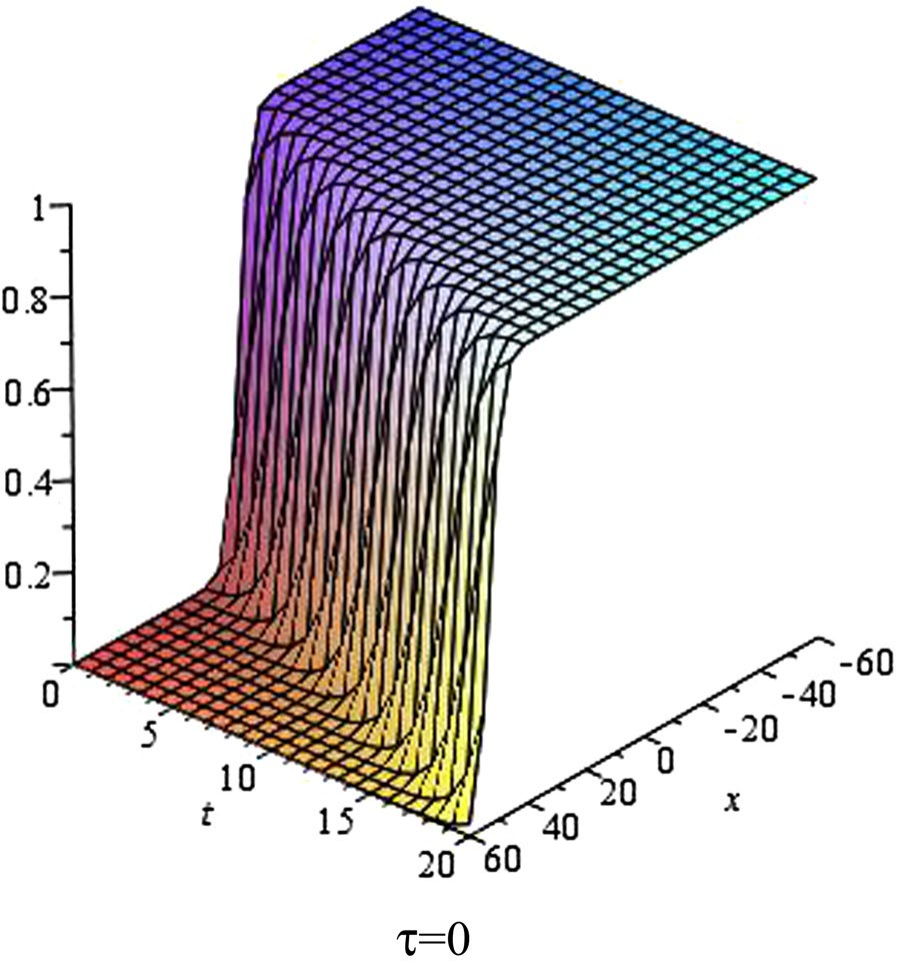
The solution (27) for $\tau = 0$ at $p=q=s=1$, $\alpha = 5$, $\beta = 4$, $t = 1$, $C_{2} = 0$

**Fig. 6:**
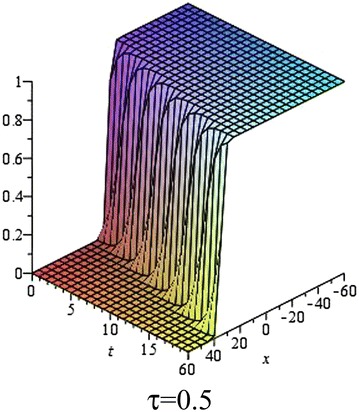
The solution (27) for $\tau = 0.5$ at $p=q=s=1$, $\alpha = 5$, $\beta = 4$, $t = 1$, $C_{2} = 0$

**Fig. 7:**
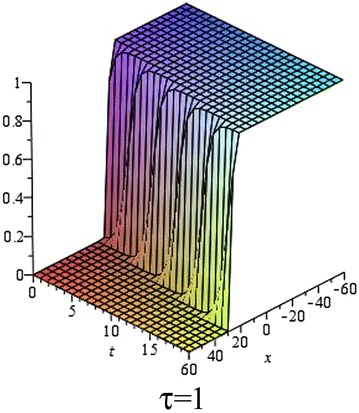
The solution (27) for $\tau = 1$ at $p=q=s=1$, $\alpha = 5$, $\beta = 4$, $t = 1$, $C_{2} = 0$

**Fig. 8:**
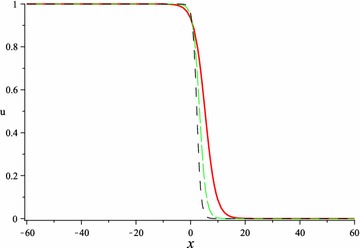
The *red*, *green* and *black lines* represent the solution (27) for $\tau = 0, 0.5, 1$ respectively at $p=q=s=1$, $\alpha = 5$, $\beta = 4$, $t = 1$, $C_{2} = 0$

In Figs. 5, 6, 7 and 8, we show the effect of the time-delayed solution (27). It should be noted that when $\tau \rightarrow 0$, we can recover some traveling wave solutions of the generalized Burgers–Fisher equation.

#### *Remark 1*

By using extended $\left( \frac{G^{\prime}}{G}\right)$-expansion method, we can obtain solutions including all the solutions given in Deng et al. (2009) as special cases. For example, if setting $C_{2}=0$, then solution (28) is the same as Eq. (19) in Deng et al. (2009). Similarly, solution (28) is also the same as Eq. (20) obtained in Deng et al. (2009) when we set $C_{1}=0$. It shows that extended $\left( \frac{G^{\prime}}{G}\right)$-expansion method is more powerful than the method in Deng et al. (2009) in constructing exact solutions.

#### *Remark 2*

Rosa et al. (2015) applied Lie classical method and $\left( \frac{G^{\prime}}{G}\right)$-expansion method to Fisher equation and derived some new traveling wave solutions. If setting $a_{l}(l=-n\ldots -1)=0$, then Eq. (4) becomes Eq. (14) in Rosa and Gandarias, (2015). So if we applied Lie classical method and extended $\left( \frac{G^{\prime}}{G}\right)$-expansion method to Fisher equation, then many more exact solutions can be obtained. Searching exact solutions by use of Lie classical method and extended $\left( \frac{G^{\prime}}{G}\right)$-expansion method is our future work.

## Conclusions

Based on the extended $\left( \frac{G^{\prime}}{G}\right)$-expansion method, we have constructed many traveling wave solutions of the time-delayed generalized Burgers-type equation which include the hyperbolic function solutions, trigonometric function solutions. The results show that the proposed method is very effective and can be used to handling many other nonlinear time-delayed evolution equations.

## Declarations

In this section, we illustrate how to get the solutions presented after Eq. (5).

The general solutions of Eq. (5) can easily obtained as follows:

$$G(\eta )= \left\{ \begin{array}{ll} a_{1}e^{\frac{-\alpha +\sqrt{\alpha ^{2}-4\beta }}{2}\eta }+a_{2}e^{\frac{-\alpha -\sqrt{\alpha ^{2}-4\beta }}{2}\eta },&\quad\alpha ^{2}-4\beta >0, \\ e^{-\frac{\alpha }{2}\eta }\left(a_{1}\cos \frac{\sqrt{4\beta -\alpha ^{2}}\eta }{2}+a_{2}\sin \frac{\sqrt{4\beta -\alpha ^{2}}\eta }{2}\right),&\quad\alpha ^{2}-4\beta <0.\\ (a_{1}+a_{2}\eta )e^{-\frac{\alpha }{2}\eta },&\quad \alpha ^{2}-4\beta =0. \end{array} \right.$$

When $$\alpha ^{2}-4\beta >0$$

$$G^{\prime}(\eta )=\frac{-\alpha +\sqrt{\alpha ^{2}-4\beta }}{2}a_{1}e^{\frac{-\alpha +\sqrt{\alpha ^{2}-4\beta }}{2}\eta }+\frac{-\alpha -\sqrt{\alpha ^{2}-4\beta }}{2}a_{2}e^{\frac{-\alpha -\sqrt{\alpha ^{2}-4\beta }}{2}\eta },$$

then

31 $$\begin{aligned} \frac{G^{\prime}(\eta )}{G(\eta )}&= \frac{\frac{-\alpha +\sqrt{\alpha ^{2}-4\beta }}{2}a_{1}e^{\frac{\sqrt{\alpha ^{2}-4\beta }}{2}\eta }+\frac{-\alpha -\sqrt{\alpha ^{2}-4\beta }}{2}a_{2}e^{\frac{-\sqrt{\alpha ^{2}-4\beta }}{2}\eta }}{a_{1}e^{\frac{-\alpha +\sqrt{\alpha ^{2}-4\beta }}{2}\eta }+a_{2}e^{\frac{-\alpha -\sqrt{\alpha ^{2}-4\beta }}{2}\eta }} \\&= -\,\frac{\alpha }{2}+\frac{\sqrt{\alpha ^{2}-4\beta }}{2}\frac{a_{1}e^{\frac{\sqrt{\alpha ^{2}-4\beta }}{2}\eta }-a_{2}e^{\frac{-\sqrt{\alpha ^{2}-4\beta }}{2}\eta }}{a_{1}e^{\frac{\sqrt{\alpha ^{2}-4\beta }}{2}\eta }+a_{2}e^{\frac{-\sqrt{\alpha ^{2}-4\beta }}{2}\eta }} \end{aligned}$$

Taking $C_{1}=\frac{a_{1}+a_{2}}{2}, C_{2}=\frac{a_{1}-a_{2}}{2},r=\frac{\sqrt{\alpha ^{2}-4\beta }}{2}\eta$, we can convert Eq. (31) into the following form:

$$\begin{aligned} \frac{G^{\prime}(\eta )}{G(\eta )} &= -\,\frac{\alpha }{2}+\frac{\sqrt{\alpha ^{2}-4\beta }}{2}\frac{(C_{1}+C_{2})e^{\frac{\sqrt{\alpha ^{2}-4\beta }}{2}\eta }-(C_{1}-C_{2})e^{\frac{-\sqrt{\alpha ^{2}-4\beta }}{2}\eta }}{(C_{1}+C_{2})e^{\frac{\sqrt{\alpha ^{2}-4\beta }}{2}\eta }+(C_{1}-C_{2})e^{\frac{-\sqrt{\alpha ^{2}-4\beta }}{2}\eta }}\\ &= -\,\frac{\alpha }{2}+\frac{\sqrt{\alpha ^{2}-4\beta }}{2}\frac{C_{1}(e^{r}-e^{-r})-C_{2}(e^{r}+e^{-r})}{C_{1}(e^{r}+e^{-r})+C_{2}(e^{r}-e^{-r})}\\&=-\,\frac{\alpha }{2}+\frac{\sqrt{\alpha ^{2}-4\beta }}{2}\frac{C_{1}\left(\frac{e^{r}-e^{-r}}{2}\right)-C_{2}\left(\frac{e^{r}+e^{-r}}{2}\right)}{C_{1}\left(\frac{e^{r}+e^{-r}}{2}\right)+C_{2}\left(\frac{e^{r}-e^{-r}}{2}\right)}\\ &= -\, \frac{\alpha }{2}+\frac{\sqrt{\alpha ^{2}-4\beta }}{2} \left( \frac{C_{1}\sinh \left(\frac{\sqrt{\alpha ^{2}-4\beta }\eta }{2}\right)+C_{2}\cosh \left(\frac{\sqrt{\alpha ^{2}-4\beta }\eta }{2}\right)}{C_{1}\cosh \left(\frac{\sqrt{\alpha ^{2}-4\beta }\eta }{2}\right)+C_{2}\sinh \left(\frac{\sqrt{\alpha ^{2}-4\beta }\eta }{2}\right)}\right) . \end{aligned}$$

When $$\alpha ^{2}-4\beta <0$$

$$\begin{aligned} G^{\prime}(\eta )&= -\,\frac{\alpha }{2}e^{\frac{-\alpha }{2}\eta }\left(a_{1}\cos \frac{\sqrt{4\beta -\alpha ^{2}}\eta }{2}+a_{2}\sin \frac{\sqrt{4\beta -\alpha ^{2}}\eta }{2}\right)\\ &\quad +\,\frac{\sqrt{4\beta -\alpha ^{2}}\eta }{2}e^{\frac{-\alpha }{2}\eta }\left(-a_{1}\sin \frac{\sqrt{4\beta -\alpha ^{2}}\eta }{2}+a_{2}\cos \frac{\sqrt{4\beta -\alpha ^{2}}\eta }{2}\right), \end{aligned}$$

then

32 $$\begin{aligned} \frac{G^{\prime}(\eta )}{G(\eta )} &= \frac{-\frac{\alpha }{2}e^{\frac{-\alpha }{2}\eta }\left(a_{1}\cos \frac{\sqrt{4\beta -\alpha ^{2}}\eta }{2}+a_{2}\sin \frac{\sqrt{4\beta -\alpha ^{2}}\eta }{2}\right)}{e^{-\frac{\alpha }{2}\eta }\left(a_{1}\cos \frac{\sqrt{4\beta -\alpha ^{2}}\eta }{2}+a_{2}\sin \frac{\sqrt{4\beta -\alpha ^{2}}\eta }{2}\right)} \\&\quad+\,\frac{\frac{\sqrt{4\beta -\alpha ^{2}}\eta }{2}e^{\frac{-\alpha }{2}\eta}\left(-a_{1}\sin \frac{\sqrt{4\beta -\alpha ^{2}}\eta }{2}+a_{2}\cos \frac{\sqrt{4\beta -\alpha ^{2}}\eta }{2}\right)}{e^{-\frac{\alpha }{2}\eta }\left(a_{1}\cos \frac{\sqrt{4\beta -\alpha ^{2}}\eta }{2}+a_{2}\sin \frac{\sqrt{4\beta -\alpha ^{2}}\eta }{2}\right)} \end{aligned}$$

Taking $C_{1}=a_{1}, C_{2}=a_{2}$, we can convert Eq. (32) into the following form:

$$\frac{G^{\prime}(\eta )}{G(\eta )}=-\frac{\alpha }{2}+\frac{\sqrt{4\beta -\alpha ^{2}}}{2} \left( \frac{-C_{1}\sin \left(\frac{\sqrt{4\beta -\alpha ^{2}}\eta }{2}\right)+C_{2}\cos \left(\frac{\sqrt{4\beta -\alpha ^{2}}\eta }{2}\right)}{C_{1}\cos \left(\frac{\sqrt{4\beta -\alpha ^{2}}\eta }{2}\right)+C_{2}\sin \left(\frac{\sqrt{4\beta -\alpha ^{2}}\eta }{2}\right)}\right)$$

When $$\alpha ^{2}-4\beta =0$$

$$G^{\prime}(\eta )=\left[a_{2}-(a_{1}+a_{2}\eta )\frac{\alpha }{2}\right]e^{-\frac{\alpha }{2}\eta },$$

then

33 $$\begin{aligned} \frac{G^{\prime}(\eta )}{G(\eta )} &= \frac{\left[a_{2}-(a_{1}+a_{2}\eta )\frac{\alpha }{2}\right]e^{-\frac{\alpha }{2}\eta }}{(a_{1}+a_{2}\eta )e^{-\frac{\alpha }{2}\eta }} \\ &= -\,\frac{\alpha }{2}+\frac{a_{2}}{a_{1}+a_{2}\eta } \end{aligned}$$

Taking $C_{1}=a_{1}, C_{2}=a_{2}$, we can convert Eq. (33) into the following form:

$$\frac{G^{\prime}(\eta )}{G(\eta )}=-\frac{\alpha }{2}+\frac{C_{2}}{C_{1}+C_{2}\eta }$$
